# Supplementary material for: Cardiovascular outcomes in adult patients with atrial septal defect: a nationwide population-based study
Source: Front Cardiovasc Med. 2025 Sep 1;12:1633002. doi: 10.3389/fcvm.2025.1633002 (PMC12434082; doi:10.3389/fcvm.2025.1633002)

Supplementary Table 1. ICD-10 codes

|  | **No procedure** | **Surgery** | **Device closure** | **Total** |
| --- | --- | --- | --- | --- |
|  |  |  |  |  |
| Atrial septal defect (Q211) | 7492 (57.2%) | 2636 (62.4%) | 530 (16.0%) | 10658 (51.6%) |
| Atrial septal defect, unspecified (Q2119) | 3870 (29.5%) | 1061 (25.1%) | 1970 (59.4%) | 6901 (33.4%) |
| Other atrial septal defect (Q2118) | 1704 (13.0%) | 514 (12.2%) | 817 (24.6%) | 3035 (14.7%) |
| Sinus venosus defect (Q2111) | 37 (0.3%) | 11 (0.3%) | 1 (0.0%) | 49 (0.2%) |

Atrial septal defect surgeries: O1710 and O1711

Atrial septal defect device closure: OZ751.

Hypertension: "I10","I11","I12","I13","I14","I15" + prescription of Hypertension medications

Diabetes mellitus: "E10""E11","E12","E13","E14" + prescription of DM medications

Hyperlipidemia: "E78"

Atrial fibrillation: "I48.x"

Pulmonary Hypertension: "I27.0", "I27.2"

Congestive heart failure (CHF): "I110","I130","I132", "I50"

Endocarditis: "I33"," I38","I39"

Stroke: "I60","I61","I62","I63"

Ischemic stroke: "I63"

SAH: "I60"

ICH: "I61","162.9"

SDH: "I62.X" (except I62.9)

MI: "I21","I22"

Coronary revascularization = I20 + procedure code.

| Procedure | ICD-10 code |
| --- | --- |
| Percutaneous coronary artery angioplasty | M6551, M6552 |
| Percutaneous coronary artery stent omplantation | M6561, M6562, M6563, M6564 |
| Percutanous transluminal coronary  angioplasty | M6571, M6572 |
| Percutaneous thrombectomy | M6633, M6634 |
| Arterial bypass graft | O1641, O1642, O1647 |
| Off-pump coronary artery bypass surgery | OA641, OA642, OA647 |

Supplementary Table 2. Hazard ratios for each cardiovascular event during the 5-year follow-up

|  | **No. of event** | **Person-years** | **Incidence rate** | **crude HR (95% CI)** | **adjusted HR (95% CI)** |
| --- | --- | --- | --- | --- | --- |
| No procedure  (n = 5,980) | 389 | 23,749 | 16.38 | (ref.) | (ref.) |
| Surgery  (n = 2,729) | 236 | 11,363 | 20.77 | 1.3 (1.100–1.530) *** | 1.34 (1.140–1.580) *** |
| Device closure  (n = 3,004) | 116 | 11,113 | 10.44 | 0.61 (0.500–0.760) *** | 0.64 (0.520–0.780) *** |

Supplementary Table 3. Hazard ratios for AF during the 3-month period following cardiac procedures

|  | **No. of event** | **Person-years** | **Incidence rate** | **crude HR (95% CI)** | **adjusted HR (95% CI)** |
| --- | --- | --- | --- | --- | --- |
| Surgery  (n = 2,729) | 117 | 528 | 221.59 | (ref.) | (ref.) |
| Device closure  (n = 3,004) | 40 | 590 | 67.8 | 0.31 (0.210–0.440) *** | 0.30 (0.210–0.430) *** |

Supplementary Figure 1. Five-year event-free survival curves for stroke subtypes


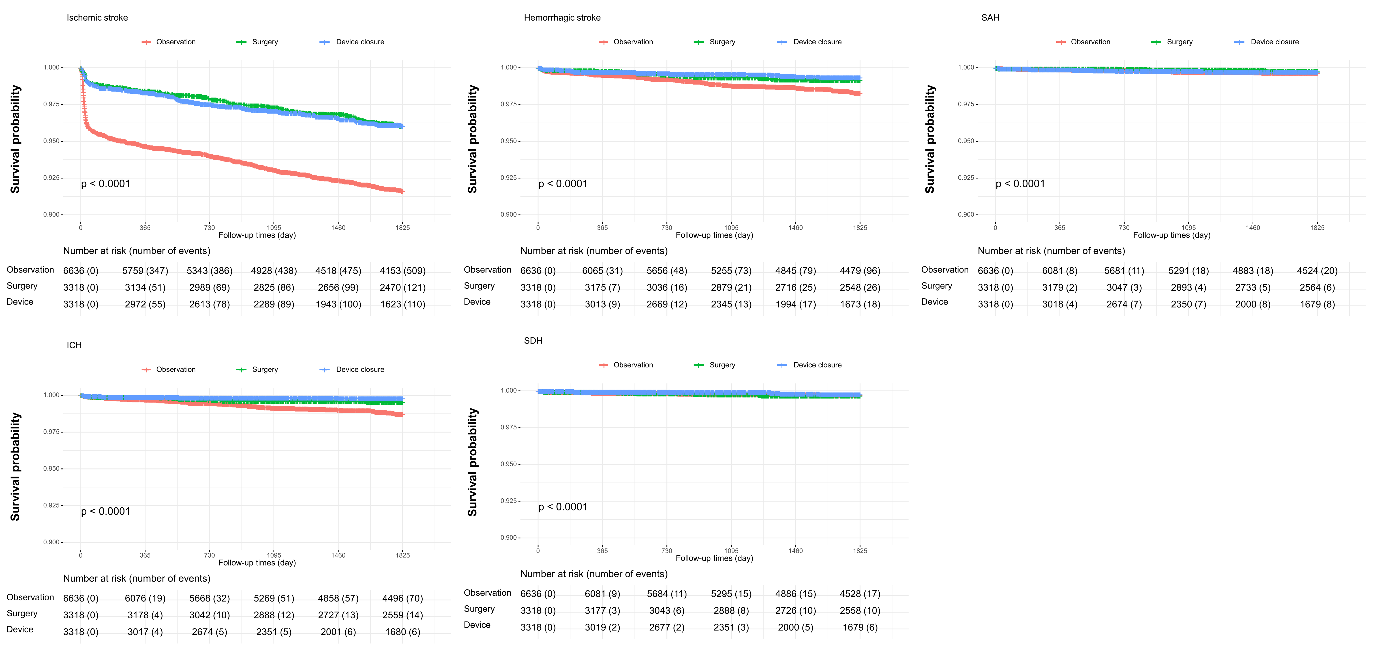

Supplement: Supplementary file 1 [file Datasheet1.docx]
